# Earthquake loss estimation of residential buildings in Bantul regency, Indonesia

**DOI:** 10.4102/jamba.v11i1.756

**Published:** 2019-06-20

**Authors:** Mohamad F.N. Aulady, Toshio Fujimi

**Affiliations:** 1 Department of Architectural and Civil Engineering, Kumamoto University, Kumamoto, Japan; 2 Department of Civil Engineering, Institut Teknologi Adhi Tama Surabaya, Surabaya, Indonesia; 3 Graduate School of Science and Technology, Kumamoto University, Kumamoto, Japan

**Keywords:** seismic vulnerability, risk curve, economic losses, casualties, hypothetical policy, Bantul regency

## Abstract

Bantul regency in 2006 had experienced considerable earthquake and suffered many casualties. The factors such as high population density and lack of seismic design of residential buildings in Bantul besides its location in a high seismic region have increased its vulnerability to earthquake disasters that can lead to a widespread economic losses and casualties. This research aims to capture earthquake risk in Bantul towards economic losses and casualties by using risk curve. Risk curve is a combination of several sources from literatures containing hazard curve and vulnerability curve together with exposure. The result showed that the expected economic loss in 50 years for residential building is $647.22 million; however, the highest value of economic losses shows the value up to $7600m which occurs in earthquake of 7.15 M_W_ scale. The same worst-case scenario caused the casualties up to 49 000 people at night-time and 15 000 people at daytime. The result established that confined masonry building type conduces the highest value of economic losses and timber frame building shows the highest vulnerability to the earthquake disaster than other building types. Furthermore, in order to reduce the risk, we applied the hypothetical policy to build a simple earthquake-resistant house called Simple Instant Healthy House. The result indicates that this mitigation policy can effectively reduce both economic losses and casualties.

## Introduction

Indonesia is an archipelago country in Southeast Asia, with a population of approximately to 261.1 million people in 2016 (World Bank 2016). Indonesia is surrounded by three tectonic plates: Indo-Australian plate, Eurasian plate and Pacific plate. Consequently, various types of disasters such as earthquake, tsunami, volcano and landslide have occurred in Indonesia (Babault et al. 2018). Yet, earthquake is one of the most life-threatening hazards in Indonesia. Earthquake is the second most oftentimes natural disaster in Indonesia, and the average number of intervals between two earthquake disasters in Indonesia was 167.77 days (Parwanto & Oyama 2014). It has been recorded that there were 246 major earthquakes from 1900 to 2012 (Parwanto & Oyama 2014). Earthquake disasters related to exposure and vulnerability all cause the economic losses (Hallegatte & Przyluski 2010). Therefore, disasters always cause a huge number of economic losses as well as casualties (Hallegatte & Przyluski 2010; Daniell et al. 2012). In 2005, there were seven earthquake occurrences that caused 1305 deaths (Parwanto & Oyama 2014). In 2006, 10 earthquake occurrences caused 5757 deaths and economic losses of $3.1 billion (Java Reconstruction Fund 2007; Parwanto & Oyama 2014). These enormous losses need to be reduced in the future by some effective prevention actions and mitigation measures. Earthquake risk mitigation and prevention have important role to understand how structures collapse because of strong ground motions, and how to classify weak structures from strong ones, and how to make the weak and bad structures stronger (Pribadi, Kusumastuti & Rildova 2008), especially for non-engineered structures which caused the most destruction during the earthquake in Indonesia, such as reinforced masonry walls, non-engineered reinforced concrete (RC) buildings and low-rise timber-framed buildings (Saatcioglu, Ghobarah & Nistor 2006).

For implementing mitigation measures effectively, we have to estimate how much earthquake risk exists in current situation, and then, how much earthquake risk can be reduced by the mitigation measure. However, there are little studies to estimate earthquake risks in terms of economic loss and causalities in Indonesia. In 2014, Ikhwan and Kusrini (2014) developed macroeconomic loss modelling because of natural disasters. They report that the independent variables that affect one region are the number of labour and disaster events. Gumila et al. (2007) investigated potential economic losses because of land subsidence in some parts of Indonesia. This study reports that land subsidence can cause high economic losses as well as economic growth in the affected area. Asih, Sumarno and Sianturi (2015) developed loss estimation model during eruption in agricultural sector. Purnama et al. (2015) estimated the economic losses and risk because of rob flood disaster. From these papers, it is noticed that no published research has estimated economic losses and casualties because of earthquake disaster in Indonesia. Therefore, this research aims to develop a procedure to capture earthquake risk by using risk curve in earthquake-prone area in Indonesia. Risk curve can express overall seismic risk with regard to the possible scenarios with occurrence probabilities, which is a plot of specific damage degree as the occupation of occurrence probability (Yoshikawa 2013). Risk curve is reliable to compare the physical building damage from the historical damage to their corresponding empirical return period. Furthermore, the earthquake risk curve can also be applied for the validation of hazard and vulnerability models (Raschke et al. 2014).

**FIGURE 1:**
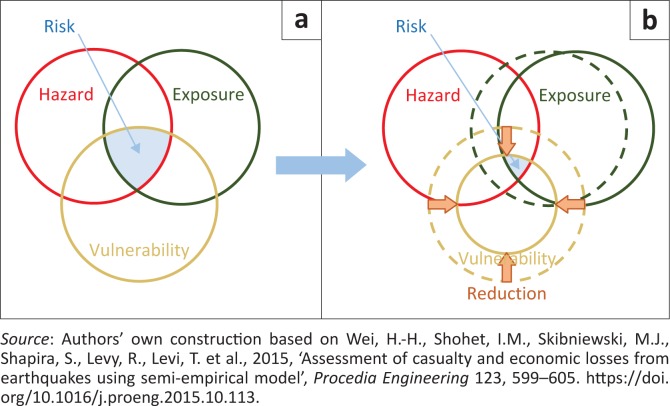
Risk assessment model: (a) normal condition and (b) after the risk reduction.

We will combine studies on hazard, vulnerability and exposure scattered in various literature to produce risk curve. On the other hand, vulnerability plays important role to capture the risk because a building which has a fairly high vulnerability becomes a major impact of fatalities in an earthquake disaster. While exposure is an accessible and approximate estimation of the element at risk studied in a given system and geographic area, exposure analysis starts from quantification of the number of factors within each vulnerability class, the number of losses and indirect losses that give damage scenarios (Cabal, Coulet & Erlich 2012). Vulnerability and hazard curves developed based on other studies are used for risk calculation, which is although reasonable approximation considering for both the existing and proposed constructions. The risk reduction by combining the study on hazard, vulnerability and exposure can be illustrated in Figure 1.

Furthermore, we chose Bantul regency because it is located in south of Java Island, under Yogyakarta Special Region Province, which is prone to earthquake disaster. It is because of subdural zone activity which is formed from collision between Indo-Australian plate and Eurasian plate in the south of Java. Moreover, there are two main factors that cause the severe damage in Bantul earthquake. Firstly, the population density of Bantul regency is so high, in 2015, it reached 1917 people per km^2^ (Statistic of D.I. Yogyakarta 2018). Secondly, Bantul regency is shortcoming of seismic design of residential buildings (Saputra et al. 2017). Thus, it will increase the possibility of fatalities and casualties because of earthquake significantly.

## Methods

### Risk curve

**FIGURE 2:**
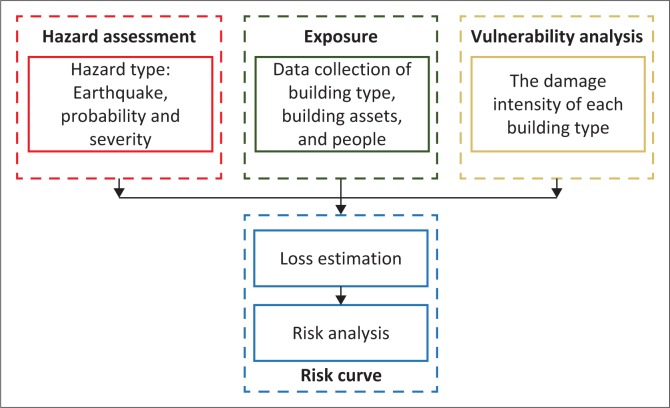
Risk curve flowchart.

This article represents earthquake risk by risk curve. Risk curve is a two-dimensional plot of authentic or projected financial risk which shows a display of correlation between probabilities of exceedance represented in vertical axis versus actual and expected financial reward (horizontal axis). The risk curves show how often the occurrence of an event such as earthquake may have consequences towards the economic loss and casualties which indicate the level of loss with different return periods. The risk curve is generally applied by decision-makers such as physical planners and civil protection institutions that allow for the instant sum of risks involved in a particular effort, making it very easy to use as a decision-making tool (Habegger 2008).To capture the risk in terms of expected economic loss and number of casualties, a combination of several sources from literatures containing hazard and vulnerability curve together with exposure in Bantul regency has been applied in risk curve, as described in Figure 2.

The first step to establish risk cure consists of hazard assessment to know earthquake situation by hazard curve and followed by identification of the element exposed by earthquake risk. The second step is exposure analysis which calculates earthquake probability of damage for each hazard scenario and for each building type. Then, the next step is vulnerability analysis based on vulnerability curve that is related to degree of damage for each type of building. The curves are generated from past event damage assessment. Furthermore, we multiply the replacement value of building damage and vulnerability to calculate losses, and then we sum up the building losses for the same hazard scenario to produce the risk curve.

### Hazard assessment

Hazard assessment is first established by hazard curve estimated by plotting annual rate of exceedance versus ground motion. It represents a correlation of occurrence probability of an incident in an area for a period of time. Hazard curve represents seismic hazard resulting from a combination of the ground motion relation and seismic hazard from fault sources: characteristic, Gutenberg Richter and seismic (Somerville 2000).

**FIGURE 3:**
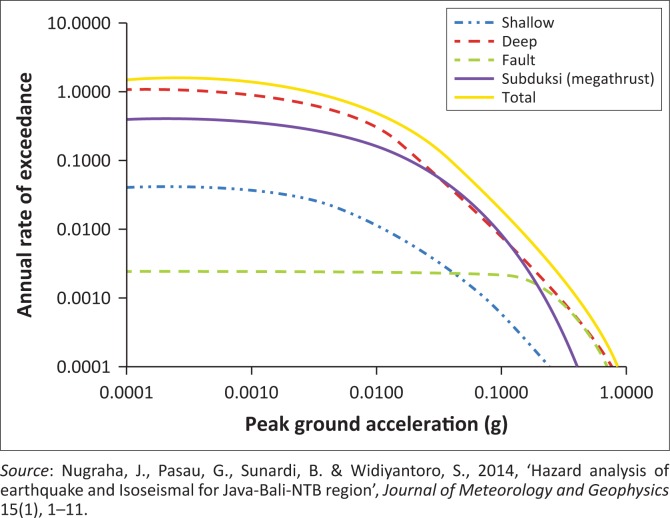
Hazard curve.

Nugraha et al. (2014) established the hazard curve of Yogyakarta province where Bantul regency is located as depicted in Figure 3. There are four lines in the curve where each line represents the earthquake source model calculation using a combination of attenuation formula. The yellow solid line is seismic hazard curve resulting from a combination of all sources. The blue dashed-dot line represents the shallow background of earthquake source whose depth is up to 50 km, the dashed red line represents the deep background of earthquake source whose depth is more than 50 km, the dashed green line represents fault earthquake source which is the terrestrial zone that occurs at clearly defined fault lines and the purple line represents sub-duction earthquake source which occurred near the encounter boundary among the oceanic plates that dip into the bottom of the continental plate. Earthquake source which has the highest hazard value of Bantul regency is fault source as the impact of Opak fault. Another source that releases the significant hazard is deep background source. The earthquakes scenario is estimated by interpolating and deriving the value of peak ground acceleration (PGA) versus various probability of exceedance in 50 years. Table 1 shows this according to solid yellow line hazard curve for the Bantul region.

**TABLE 1:** Peak ground acceleration derived from hazard curve of Bantul regency for seven earthquake scenarios.

Probability of exceedance in 50 years (%)	Value of probability on graph	PGA (g)	Intensity
100	2 × 10^−2^	0.10	6.49
50	1 × 10^−2^	0.16	7.15
10	2 × 10^−3^	0.32	8.08
5	1 × 10^−3^	0.41	8.44
2	2 × 10^−4^	0.58	8.85
1	2 × 10^−4^	0.74	9.18
0.5	1 × 10^−5^	0.86	9.36

PGA, peak ground acceleration.

### Exposure

To determine the effect of the hazard, the first step is analysing and reconstructing the affected environment. In most cases, exposure data identify various types of physical land entities, including building assets, infrastructure, agricultural land and people. Based on the number of residential dwellings and population living in Bantul which were obtained through the literatures and public statistics, a sampling in the target area and performing questionnaire survey distinguishes the percentage of various building typologies, the number of inhabitants in each type dwellings, cost value of the house and other necessary data for loss estimation (Sengara et al. 2010). The sampling is based on the Slovin equation, which is generally used for the huge sample, and this formula determines the smaller sample that can represent the whole data (Yamane 1967).

Equation 1 (Yamane 1967):

$$n=\frac{N}{1+Ne^{2}}$$ [Eqn 1]

where,

*n*: sample size

*N*: population size.

**FIGURE 4:**
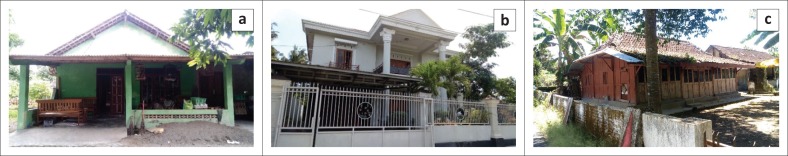
(a) Sample of confined masonry, (b) sample of reinforced concrete medium rice frame with masonry infill walls and (c) timber frame.

From the questionnaire survey, we get the number of each building type, building topology, building price, the assets and the number of family member of each building in Bantul regency. According to survey result, we classify the building type of Bantul regency into five types of buildings: unreinforced masonry (UM), RC low rice frame with masonry infill walls (RCLRFM), confined masonry (CM), RC medium rise frame with masonry infill walls (RCMRFM) and timber frame (TF). The total number of building in Bantul regency is 369.106. The highest number of building type is CM building, as depicted in Figure 4a, which has 198 919 buildings and it is occupied by five people of each building. The average price of CM building is $14 000.00. The lowest number of building type is RCMRFM for three storeys, as depicted in Figure 4b, which has 1105 buildings and it is occupied by eight people of each building, and the average price of building is $126 000.00. Moreover, TF building type which is depicted in Figure 4c is typically the cultural and historical building in Bantul area constructed from wood or timber which is called the ‘Joglo house’.

Most of the building topology is residential building, because most of the population is farmer which is 25% of all population in Bantul regency. Table 2 presents the summary of questionnaire survey in Bantul region.

**TABLE 2:** Sample statistic of target area.

No	Building type	Symbol	Description	Percentage of buildings	Average building price (thousand USD)	Average building assets (thousand USD)	Average member living at daytime (people)	Average member living at daytime (people)
**1**	Unreinforced masonry	UM	Constructed from brick, with no columns or beams, roofs made of tiles or asbestos	18.86	7.00	2.25	2	4
**2**	RC low rise frame with masonry infill walls	RCLRFM	The building has a frame (beams and columns) constructed from concrete. The frame is built first, and then it is continued for the wall	3.59	14.00	6.29	2	4
**3**	Confined masonry	CM	An improved version of UM, widely used by Indonesian people. The construction between walls and columns is done almost simultaneously	53.89	14.00	6.39	2	4
**4**	RC medium rise frame with masonry infill walls	RCMRFM	Constructed from frame concrete for buildings with medium height	14.97	126.00	29.98	3	6
**5**	Timber frame	TF	Constructed from timber	8.68	14.00	6.16	2	4

RC, reinforced concrete; UM, unreinforced masonry; RCLRFM, reinforced concrete low rise frame with masonry infill walls; RCMRFM, reinforced concrete medium rise frame with masonry infill walls; CM, confined masonry; TF, timber frame; USD, United States dollar.

### Vulnerability curves

**FIGURE 5:**
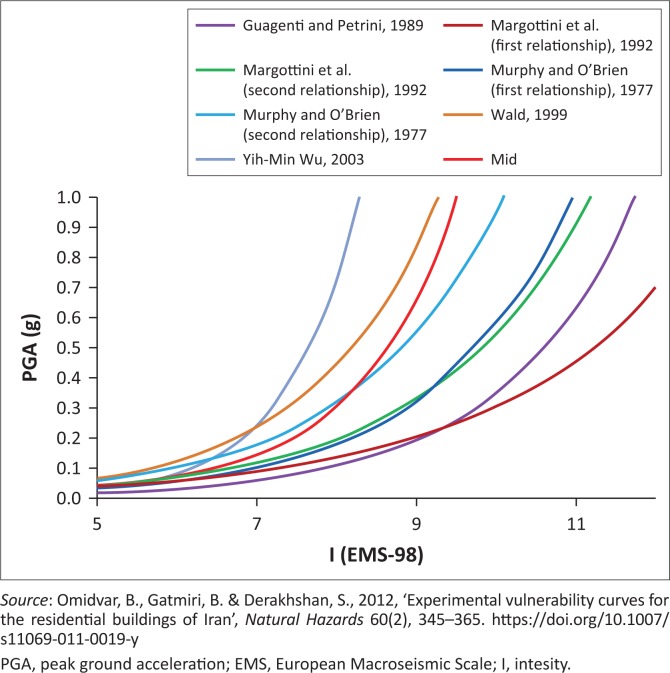
Different correlation between peak ground acceleration and intensity.

Vulnerability curves define the physical vulnerability as a function of the process intensity and the level of loss, reflecting some structural characteristics of the affected buildings (Papathoma-Köhle 2016; Sengara et al. 2010). Figure 5 shows some correlations between PGA and intensity (*I*) in European Macroseismic Scale (EMS-98) based on different sources. Table 3 shows damage index table derived from vulnerability curve for residential building typologies in Padang, Indonesia, which typically has the same building typology as Bantul regency. As depicted in Figure 5, the red line which is the mid curve has been applied to interpolate the value of intensity based on the value of PGA which derived from hazard curves of Yogyakarta. Then, the value of damage ratio for those building types which exist in Bantul regency is derived from vulnerability curve for each intensity. Damage ratio of buildings is the ratio between the repair cost of the building to construction cost. By an average of total cost of each building types and properties inside the building which were the results of questionnaire survey in Bantul regency, we then estimated the economic loss. For casualty’s estimation, we use a model developed by Coburn and Spence (2002).

**TABLE 3:** Damage index derived from vulnerability curve from the Padang earthquake damage observations.

No	Symbol	Building typologies	Intensity						
			6.49	7.15	8.08	8.44	8.85	9.18	9.36
1	UM	Unreinforced masonry	0.00	0.02	0.20	0.36	0.54	0.69	0.74
2	RCLRFM	RC low rise frame with masonry infill walls	0.00	0.01	0.08	0.23	0.43	0.62	0.71
3	CM	Confined masonry	0.00	0.00	0.07	0.24	0.48	0.69	0.77
4	RCMRFM	RC medium rise frame with masonry infill walls	0.00	0.00	0.22	0.55	0.86	0.96	0.98
5	TF	Timber frame	0.00	0.01	0.13	0.25	0.42	0.56	0.62

UM, unreinforced masonry; RCLRFM, reinforced concrete low rice frame with masonry; RCMRFM, reinforced concrete medium rise frame with masonry infill walls; CM, confined masonry; TF, timber frame; USD, United States dollar.

Based on the vulnerability curve, the history of Bantul earthquakes indicated the similar trend to those observed in Padang which shows the poor performance of CM, which is a deficiency of structural integrity of various elements in the building. These issues created separations of structural components and imperfect collapse, or absolute collapse. Additionally, the most damages occurred on the walls, as these elements were often left unconnected to beams and columns. Many buildings are designed with structural irregularities (Pribadi et al. 2008). This structure frequently does not comply the minimum requirements on superior CM buildings, and some of them use locally available materials to give a ‘masonry-like’ features; in fact, the vulnerability to the ground shaking is high (Boen & Pribadi 2007). Regarding the TF types, it has the lowest performance because of the structural resources that are very prone to the ground shaking.

## Result

**FIGURE 6:**
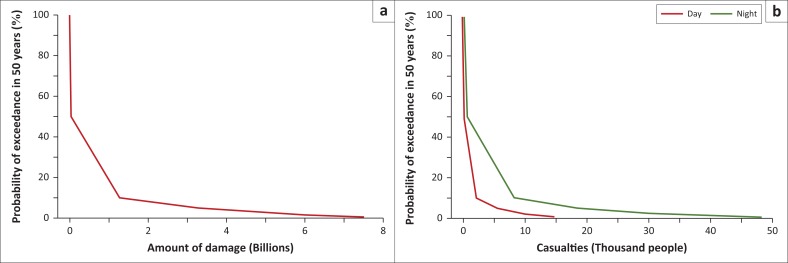
Calculation of risk in terms of (a) economic loss and (b) casualties for all residential building in Bantul region.

Figure 6 depicts risk curve in terms of economic loss and casualties for all residential buildings in Bantul region. A large area under the risk curve in Figure 6(a) shows a huge increase in economic loss for the scenario of 50% probability of exceedance in 50 years and lower probabilities. In this level, an earthquake magnitude of 5.86 or greater on the M_W_ scale will cause economic loss and casualties significantly. Area under risk curve describes the expected economic loss in 50 years for residential building, which is $647.22m; however, the highest value of economic losses shows the value up to $7600m which occurs in earthquake of 7.15 M_W_ scale.

The same worst-case scenario in earthquake magnitude of 7.15 causes the casualties up to 49 000 people at nigh-time and 15 000 people at daytime, as shown in Figure 6b. These values are equal to 4.91% and 1.50% of population in Bantul region. The expected casualties’ number in 50 years is 1122 and 4115 people for daytime and night-time, respectively.

**FIGURE 7:**
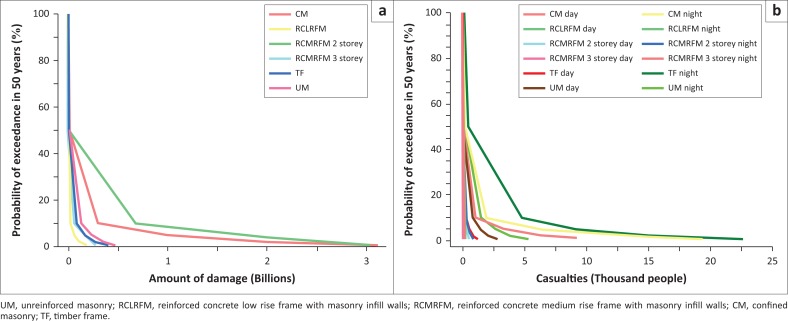
Established that risk curves for each building type in terms of (a) economic loss and (b) casualties.

To apply for government support, we will derive the economic losses and casualties’ calculation into the risk structure in detail (each building type), as depicted in Figure 7. The concaved-shaped curve observed in all curves describes that the smaller the probability exceedance, the greater the damage.

The result indicates that CM buildings type conduces the highest value of economic loss which is approximated to $3123m for the worst-case scenario, as shown in Figure 7(a). This value is followed by RCMRFM building type which causes economic losses up to $2811m. On the other hand, TF building type takes the fourth value for economic losses. However, in terms of casualty amount as shown in Figure 7(b), TF building causes the highest value of casualties which are 1173 people at daytime and 22 638 people at night-time. The second highest number of casualty is caused by CM buildings which are 10 282 people at daytime and 19 385 people at night-time. This indicates that TF and CM buildings have higher vulnerability to the earthquake disaster than other building types.

## Discussion

There are two important results in this research. Firstly, CM building produces the highest economic losses followed by RCMRFM building type. It is because CM building is the most widely used building in Bantul regency, accounting for more than 50% of all building types in the area. RCMRFM building, which is the most expensive one, takes the third place of the building used in Bantul regency. Besides, TF building, which is the fourth highest, results in the economic losses because of the lowest price of the building; moreover, only 8.19% families in Bantul regency are using this type of construction.

Secondly, in terms of casualties’ result, TF building conduces the highest value of casualties. Timber frame structures are earthquake-resistant because of the dissipative capacity of the joints and their ability to dissipate large amounts of energy (Lukic et al. 2018). Yet, TF structure experienced the flexural mechanism of ground shaking and it decreases the structure of the building (Lukic et al. 2018). In the case of Bantul area, TF buildings are typically Joglo (Javanese wooden) house which the construction technique of roof type is futuristic and complex. Structural proportion, especially the joints, plays the critical role against the seismic vulnerability. Even though TF building type is well known to be resistant to the major earthquake, the load-carrying property of a complex traditional joint on Joglo house has not clarified very well. The dimensional proportion of the joint at the main column and its position in height does not follow the seismic building code, yet it follows traditional carpenter’s common rule. The initial slackness of the joint leaded to the larger deformation. Thus, TF building in Bantul regency has high vulnerability to the earthquake disaster compared to other building typologies (Prihatmaji, Kitamori & Komatsu 2014, 2015). Furthermore, CM building causes the second highest of casualties, although CM structures usually were excellent in resisting past earthquakes. However, it was observed that CM walls with openings typically experience more significant damage, especially when the confinement of CM building is inadequate (Alcocer, Arias & Vázquez 2004; Singhal & Rai 2013; Yáñez et al. 2004). If the opening of CM wall is considered to be large, it then significantly influenced the stress distribution and resulting lateral stiffness of the wall (Yáñez et al. 2004). The typical CM building in Bantul area by our survey has a large opening wall.

According to the Figure 6, 10% probability exceedance in 50 years is equal to 90% of risk value or 90% probability of damage value which is amounted to be $1267.47m. In other words, the minimum cost that needs to be pay out by government for recovery of 90% probability is $1267.47m. The economic loss of 95% and 99% value at risk would be $3288.18 and $7034.47m, respectively. Meanwhile, the casualty number of 90% value at risk is closed to 2073 people at the daytime and 8164 people at night-time. Then, the casualty number of 95% value at risk would be 5305 people at daytime and 18 293 people at night-time, respectively. Yet, the increasing value at risk from 95% to 99.5% is much higher than from 90% to 95%. As we compared to the economic losses of post disaster records (Rosyidi et al. 2008), the losses are approximately to $1064.45m for residential building which occurred on earthquake magnitude of 6.3 or equal to 90% value at risk. Moreover, the number of casualties recorded was 23 524 people at night-time (05:55) (Murakami, Pramitasari & Ohno 2008). The calculation of this study has slightly different from real data, indicating that risk curve method can be effectively applied for loss reduction measure of earthquake disaster.

**FIGURE 8:**
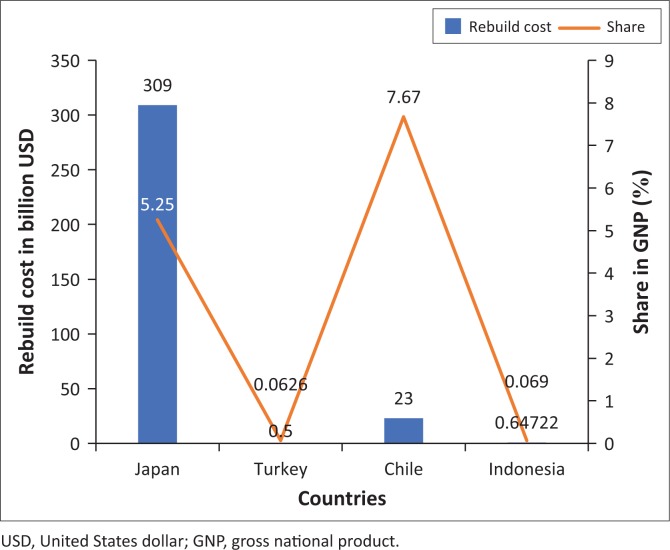
The comparison of rebuild cost and share in gross national product (%) from different countries.

This economic loss calculation can be applied by Indonesian government or local government to allocate the disaster risk management (DRM) budget to rebuild the damage building after earthquake disaster. From Figure 6, the expected value of economic losses shown is closed to $647.22m, which means that the minimum cost to rebuild the building after earthquake is $647.22m. However, according to gross domestic product of Bantul City in 2014 which is $348.98m, it is still inadequate for Bantul government to support residents. Given that this earthquake disaster is pertained to be a very serious disaster, it is not only local government but also the Indonesian government that needs to support residents. If it is compared to the national budget, the expected value of economic losses is 0.069% of gross national product (GNP) of Indonesia in 2016. It is still not sufficiently large for the Indonesian government to rebuild the damage building after earthquake disaster. Moreover, if we compare with data from other countries that have very high allocation budget for earthquake DRM, such as Japan, Turkey and Chile, the rebuild costs are 5.25%, 0.0626%, and 7.67% of GNP, respectively (Platt & So 2017) which is described in Figure 8 in detail.

This comparison indicates that Indonesia has not been applied the earthquake DRM effectively. Moreover, considering this high number of economic losses and casualties, the disaster risk needs to be reduced. Hence, this study intends to apply some policy to reduce the disaster risk as explained in the following section.

## Policy implication

**FIGURE 9:**
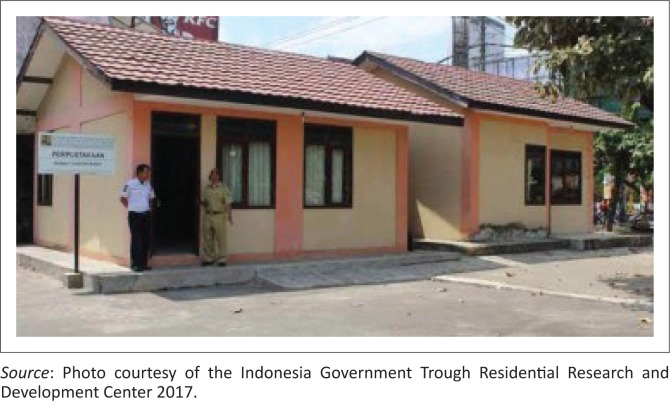
Example of Simple Instant Healthy House.

To reduce the risk in future earthquake, we intend to simulate a hypothetical mitigation policy. As it was indicated in previous section, the numbers of CM and TF dwellings play a crucial rule to increase the risk. In this section, a policy containing replacement of a number of CM and TF dwellings to engineering-based reinforced concrete house called Simple Instant Healthy House (RISHA) (Figure 9) has been enforced as.

Simple Instant Healthy House is the embodiment of a modular design, a concept that divides the system into small parts (modules) of efficient sizes in order to be assembled into a large number of different products (Sabaruddin & Sukaman 2015). Simple Instant Healthy House has been launched by the Indonesian government through Residential Research and Development Centre under the Ministry of Public Works and Residence. Simple Instant Healthy House construction cost for an area of 40 m^2^ (type 36) is $6332.23. Residential Research and Development Centre claims that RISHA is resistance until eight richer scalars or eight in Mercalli Modify Intensity scalar. However, RISHA has still less public interest because of the lack of promotion; this is the reason why so many people do not know about RISHA. So far in Yogyakarta, the enthusiasts are still dominated by the government. In 2014, there were only 16 units, and it is increased only five units in 2015 (Indonesia Government through Residential Research and Development Center 2017)

From our observation and the literature studies, there are several factors why RISHA has not attracted public interest. Firstly, RISHA is not very popular in the community. In fact, people’s interest in cheap and healthy housing is so high. Secondly, RISHA has not attracted developer attention to build this house. The developers can be the media to further introduce RISHA and to meet the RISHA for residential building as residence requirement. Thirdly, Bantul community seems to be unfamiliar with the knock-down and instant system of RISHA building. Thus, this makes the uncertainty of the community to build the RISHA. Fourthly, from the literature survey, some people (40% from 107 respondents) feel that the area of RISHA building type 36 needs to be expanded and the openings and ventilation needs to be increased (Heston 2015). Therefore, this research intends to apply the hypothetical policy which RISHA can be used for government programme to reconstruct the damage building after Bantul earthquake. We believe that this programme will increase the public interest to apply the RISHA. Moreover, RISHA can decrease the economic losses and casualties in the future.

The policy is applied to reconstruct the 10% of CM and TF buildings by RISHA. The economic losses and casualties are subsequently recalculated after 10% of CM and TF buildings are replaced by RISHA. The hypothetical policy consists of two procedures which are the reconstruction by the homeowner or by government subsidy. To engage a high degree of risk reduction to transform earthquake vulnerability to earthquake resistance in Bantul regency, a 100% subsidy by government to replace the damage building with RISHA has been applied.

Later, the total cost of policy is compared to the economic loss reduction and number of decreased casualties after mitigation policy implementation scenarios. As shown in Table 4, we assume that 100% of reconstruction expense is subsided by government for each dwelling

**TABLE 4:** Breakdown of total amount to be subsided for confined masonry and timber frame buildings.

No.	Building typology	Replaced building	Policy cost for each dwelling (USD)	10% of each typology	Total amount of policy cost (million USD)
**1**	Confined masonry	RISHA	6223.23	19 892	123.79
**2**	Timber frame	RISHA	6223.23	3205	19.94

USD, United States dollar; RISHA, reinforced concrete house called Simple Instant Healthy House.

**FIGURE 10:**
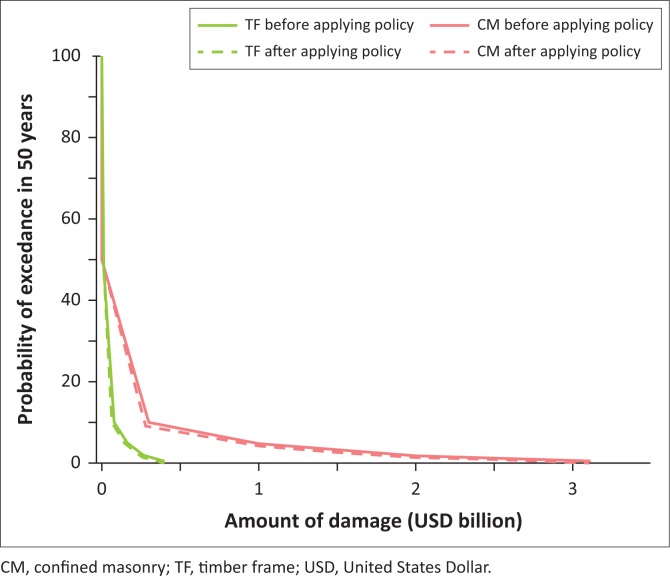
Comparison of risk calculation in terms of economic loss for confined masonry and timber frame buildings before and after applying policy.

**FIGURE 11:**
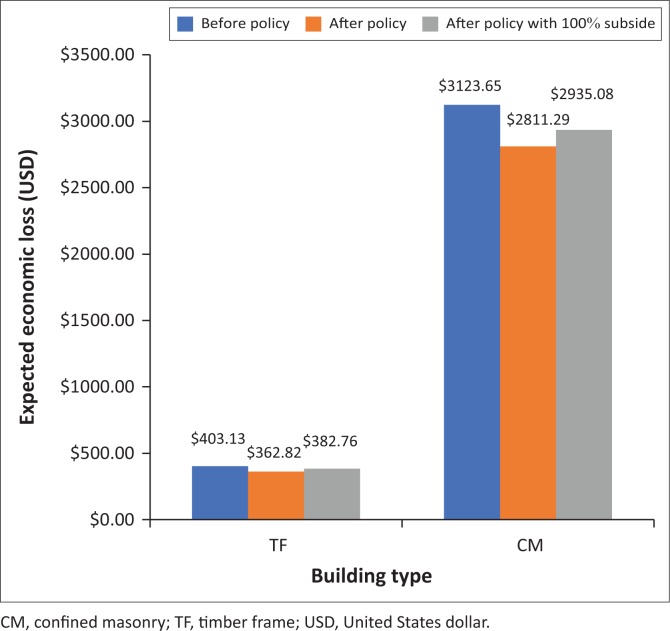
Comparison of expected economic loss for confined masonry and timber frame building before and after applying policy.

The area under risk curve which stands for expected loss in 50 years has been calculated and compared for both typologies before and after applying policy, as shown in Figure 10 and Figure 11. Based on the calculation result, the reduction number of expected economic losses of CM and TF buildings after applying policy are $312.37m and $40.31m, respectively. Surprisingly, these amounts are higher than the amount which is consumed as policy cost, indicating that the hypothetical mitigation policy is effective for economic loss reduction in both typologies.

**FIGURE 12:**
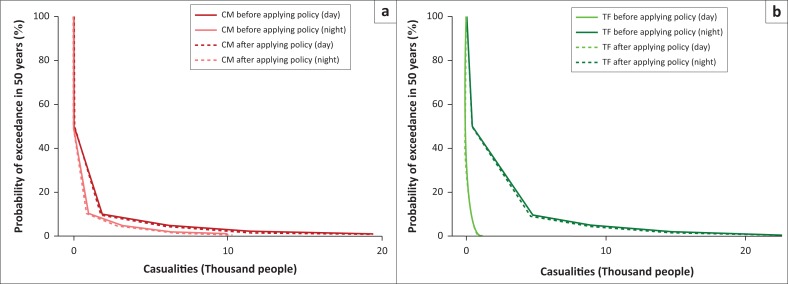
Risk curves in terms of number of casualties for (a) confined masonry (b) timber frame before and after applying policy.

**FIGURE 13:**
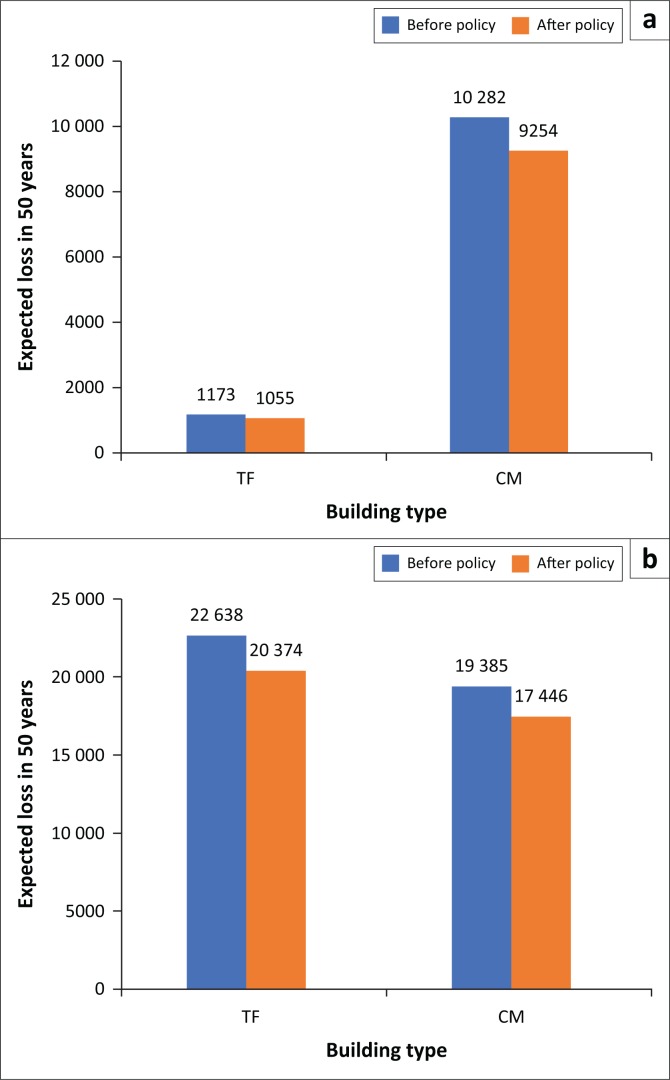
A comparison of casualty reduction in expected loss of lives for (a) daytime and (b) night-time earthquake scenario.

Figure 12 depicts the number of casualties for CM and TF buildings before and after reconstructing 10% of the building with RISHA. Figure 13 shows the comparison of expected number of casualties which indicate that by applying policy for both CM and TF buildings, the expected numbers of casualties are deceased. This reduction numbers are 1028 and 117 people in daytime earthquake scenario and 1938 and 2264 people in night-time earthquake scenario for CM and TF buildings, respectively. This result indicates that this hypothetical mitigation policy is not only effective to reduce the economic losses but also affective to reduce the number of casualties.

## Conclusion

In this study, the economic losses and casualties of Bantul earthquake in the future have been calculated and predicted. Our method using risk curve to capture earthquake risk in Bantul has resulted in how often the occurrence of Bantul earthquake has consequences towards economic loss and casualties; this indicates the level of loss with different return periods. The results established that the residential building is exposed the highest value of economic losses, CM building type conduces the highest value of economic losses and TF building shows the highest vulnerability to the earthquake disaster than other building types. Furthermore, we applied the policy to reconstruct the damage building with a simple earthquake-resistant house called RISHA to reduce the risk. The result indicates that this hypothetical mitigation policy is not only effective to reduce the economic losses but also affective to reduce the number of casualties.
